# Are Those Who Protect Protected? Occupational Risk Among Medical Students in Romania

**DOI:** 10.3390/pathogens15090931

**Published:** 2026-09-04

**Authors:** Andreea Marilena Păuna, Bianca Georgiana Enciu, Carmen Daniela Chivu, Oana Săndulescu, Maria-Dorina Crăciun, Daniela Pițigoi

**Affiliations:** 1 Department of Epidemiology I, Carol Davila University of Medicine and Pharmacy, 020021 Bucharest, Romania; andreea.pauna@umfcd.ro (A.M.P.); carmen-daniela.chivu@umfcd.ro (C.D.C.); maria.craciun@umfcd.ro (M.-D.C.); daniela.pitigoi@umfcd.ro (D.P.); 2 Department of Epidemiology II, Carol Davila University of Medicine and Pharmacy, 020021 Bucharest, Romania; 3 Department for Infection Prevention and Control, Emergency Clinical Hospital for Children “Grigore Alexandrescu”, 011743 Bucharest, Romania; 4 Department of Infectious Diseases I, Carol Davila University of Medicine and Pharmacy, 020021 Bucharest, Romania; oana.sandulescu@umfcd.ro; 5 National Institute for Infectious Diseases “Prof. Dr. Matei Balș”, 021105 Bucharest, Romania

**Keywords:** accidental exposure, medical students, prevention, training, management

## Abstract

Occupational exposure to blood and body fluids (OEBBF) continues to represent an important public health problem due to the associated medical, social and economic consequences. Young healthcare workers are at high risk of OEBBF because of their lack of experience, hesitation before performing a task and the nature of the task performed. This study aims to comparatively evaluate the knowledge, attitudes and perceptions on OEBBF among students from the Faculty of Medicine and the Faculty of Dental Medicine at Carol Davila University of Medicine and Pharmacy, Bucharest, Romania, during the 2021–2022 academic year in order to facilitate the adaptation of educational materials used in training programs. Out of the 911 students who completed the questionnaire, 55% (502) correctly identified hepatitis B virus (HBV) as having the highest risk of transmission after an OEBBF, with statistically significantly higher percentages among Dental Medicine students (152; 60% vs. 350; 53%; *p* = 0.003). However, lower percentages of Dental Medicine students recognized the protective concentration of antibodies against HBV (173; 68% vs. 536; 82%; *p* < 0.001) or reported knowing to be vaccinated against hepatitis B (110; 43% vs. 459; 70%; *p* < 0.001). Additionally, higher percentages of Dental Medicine students reported not having an antibodies concentration measurement since they started their clinical practice (206; 81% vs. 439; 67%; *p* < 0.001). If an OEBBF were to occur, 43% (391) reported that they would induce bleeding, either instinctively (210; 23%) or because they considered it necessary (181; 20%). Ninety-three students (10%) reported at least one past OEBBF; 59 (63%) reported it to their supervisors. Statistically significantly higher percentages of Dental Medicine students reported a high perceived risk of exposure compared to Medicine students (117; 46% vs. 202; 31%; *p* < 0.001), with the finding supported by univariable and multivariable analyses. In conclusion, the reporting frequency of past OEBBF in our study population was low. However, enhanced efforts are required to improve knowledge and awareness regarding OEBBF risk and preventive measures. Additionally, efforts should focus on assessing the level of protection against hepatitis B among Dental Medicine students and on promoting timely reporting to ensure appropriate post-exposure management.

## 1. Introduction

Occupational risk for healthcare workers (HCWs) is increasingly being discussed, having serious implications for healthcare systems through its associated physical, psycho-emotional, social and economic impact. Within the concept of occupational risk, infectious risk resulting from an occupational exposure to blood and body fluids (OEBBF), defined as a percutaneous injury or contact of the mucous membrane or non-intact skin with blood, tissue, or other potentially infectious body fluids, is particularly important.

Although efforts have been made to implement protocols and practices regarding standard precautions, staff training, and risk awareness, according to World Health Organization (WHO) estimates, three million HCWs are exposed to bloodborne pathogens annually, of which 170,000 are exposed to HIV infections, 500,000 to hepatitis C virus (HCV) infections and 2 million to hepatitis B virus (HBV) infections [1].

The highest risk is found among surgeons, emergency room workers, staff working in laboratories, and nurses. In addition to hospital employees, personnel working in other types of clinics and medical offices, emergency services, elderly care centers, and home care can suffer accidents, and their incidence remains unknown.

The risk of developing an infectious disease after an occupational exposure to blood or blood-containing fluids is not negligible, with over 20 infectious agents potentially involved, but the highest risk is encountered in the case of HBV, HCV, and HIV [2,3].

The risk of infection after exposure depends on the prevalence of these infections in the population, as well as on the type and frequency of exposure, with more infections occurring in sub-Saharan Africa [4].

In the EU/EEA, HBV prevalence is estimated at 0.9%, which is lower than in western and northern European countries and higher than in southern and eastern European countries, corresponding to about 4.7 million chronic HBV cases. HCV prevalence is estimated at 1.1%, corresponding to about 5.6 million chronic HCV cases. In 2023, 112,883 HIV diagnoses were reported in 47 countries in the WHO European Region, including 24,731 in the EU/EEA, giving a rate of 12.7 per 100,000 population [5,6,7].

In HCWs, needlestick injuries (NSIs) are estimated to be responsible for 37% of HBV infections, 39% of HCV infections, and 4.4% of HIV infections [8].

There are international concerns with respect to the prevention of OEBBF, including through legislation such as Council Directive 2010/32/EU of 10 May 2010 implementing the Framework Agreement on prevention of sharp injuries in hospitals and the Needlestick Safety and Prevention Act, which was adopted in the US in 2000 [8].

In Romania, all healthcare facilities and HCWs are legally required to implement preventive measures and adhere to established protocols to reduce the risk of OEBBF and its associated infectious consequences. These measures include clearly defined responsibilities, regular updates of pre-exposure and post-exposure management protocols; comprehensive and targeted staff training; and the adequate provision of personal protective equipment, sharps disposal containers, and safety-engineered devices such as needle-free and protected sharps systems. Vaccination programs, particularly against hepatitis B, are also essential. Risky behaviors should be minimized through standardized procedures and strict adherence to safety practices aimed at preventing exposure (primary prophylaxis) or reducing the likelihood of infection after an exposure (secondary prophylaxis).

According to the national guidelines, primary prophylaxis of OEBBF involves adhering to hepatitis B vaccination recommendations and universal precautions, exercising extra caution when handling sharp instruments, promptly disposing of sharp waste in secure containers, and avoiding the recapping of needles [9,10]. Secondary prophylaxis focuses on proper OEBBF case management, which is considered a medical emergency [11]. It involves temporarily halting care while ensuring patient safety, providing emergency treatment, reporting the event, collecting blood samples from both the injured person and the source patient, documenting the OEBBF case, and consulting with an infectious disease specialist [9,10].

Effective prevention and management of OEBBF risks depend also on proper monitoring and surveillance of OEBBF, with all healthcare facilities in Romania having the responsibility to record and report every occupational exposure to blood and body fluids to public health authorities [9].

According to publicly available data from the National Institute of Public Health, 1373 accidents were reported in 2024, resulting in an incidence rate of 0.4 per 100 HCWs. This represents an increase of 289 cases compared with 2023, when 1084 accidents were recorded. In 2024, OEBBF cases in Romania were most frequently reported among young healthcare workers aged 25–29 years (20%) and individuals with fewer than five years of medical experience (36%), with this data being in line with other studies showing young healthcare workers, including medical students, as being at high risk of exposure for several reasons, including lack of experience, hesitation before performing a task, the nature of the task performed, handling of biological samples, and close contact with patients [4,12].

At our university, information on professional exposure and its management is included in the curriculum of the epidemiology module. This module is scheduled in the sixth year of study for Medicine students (final year) and in the fourth year of study for Dental Medicine students, when they begin intense clinical practice.

Therefore, considering the documented high risk of OEBBF among young healthcare professionals and the limited data on its incidence and risk perception among medical and dental students in Romania, this study aims to comparatively assess the knowledge, attitudes and perceptions on OEBBF among Medicine and Dental Medicine students in order to develop targeted training recommendations tailored to the specific needs of each group.

## 2. Materials and Methods

### 2.1. Study Design

We conducted a cross-sectional study among Medicine and Dental Medicine students from the Carol Davila University of Medicine and Pharmacy, Bucharest, Romania, during the entire 2021–2022 academic year. The extensive study methodology was previously described [13]. Briefly, two electronic questionnaires were developed in Google Forms—one for Medicine students and one for Dental Medicine students. The questionnaires included similar items, all mandatory, addressing demographic characteristics, medical practice, knowledge and attitude towards vaccination, standard precautions and management of an occupational exposure to blood and body fluids.

All 1187 students enrolled in the Epidemiology course were invited to complete an online questionnaire in Romanian at the beginning of the first course. The questionnaire required about 20 min to be completed.

Data collection took place from 7 October 2021 to 31 May 2022. A total of 911 students participated, resulting in a response rate of 77%.

### 2.2. Operational Definitions

Occupational exposure to blood and body fluids: Any occupational exposure experienced by a healthcare worker that poses a risk of HIV, HBV, or HCV infection and requires post-exposure prophylaxis, including percutaneous injuries (e.g., needlestick injuries or cuts from sharp objects), contact with mucous membranes or non-intact skin (e.g., abrasions, cuts, lacerations, blisters, eczema, or other dermatological conditions), prolonged contact with intact skin (e.g., lasting several minutes or longer), or exposure of an extensive skin area to blood, tissues, or other biological materials visibly contaminated with blood [9].

Standard precautions: Minimum infection prevention measures applied to all patients receiving care in any healthcare setting, regardless of their suspected or confirmed infectious status. It includes hand hygiene, use of personal protective equipment, injection safety, proper handling of medical equipment and devices, respiratory hygiene and cough etiquette [9].

Universal precautions: Measures applied to prevent the transmission of HIV, HBV, HCV, and other blood-borne pathogens during healthcare procedures. Their basic principles include the following: all patients are considered potentially infected; blood, other body fluids, and tissues from all patients are considered potentially infectious with HIV, HBV, HCV, and other pathogens transmitted through the parenteral route; and needles and other objects used in medical practice are contaminated after use [14].

### 2.3. Statistical Analysis

For this paper we carried out a descriptive and comparative analysis of data related to the students’ knowledge (universal precaution principles, standard precaution measures, correct sequence of personal protective equipment (PPE) doffing, infectious risk associated with the exposure, protective anti-HB antibody concentration, and post-exposure management—inducing bleeding, appropriate management following an ocular mucosa exposure and event announcement) and practices with respect to OEBBF prevention (hepatitis B vaccination status, measurement of anti-HB antibody concentration, use of PPE, needle-recapping habits, and disposal of used PPE and sharp instruments). Additionally, we compared the two groups in terms of past exposures (frequency, type of exposure, reason, and if they announced the event to a supervisor) and risk perception. All analyses were exploratory.

Categorical variables are presented according to the response categories provided in the questionnaire, reporting frequencies and percentages. For perceived risk, the 5-point Likert scale was dichotomized into two categories: lower perceived risk (scores of 1–3) and higher perceived risk (scores of 4–5), allowing students with a high perceived risk to be distinguished from those reporting moderate or lower perceived risk. For continuous variables, we calculated median and interquartile ranges (IQRs).

Differences between groups were assessed using Pearson’s Chi-squared test and Fisher’s exact test for categorical variables; the Wilcoxon rank-sum test was used for continuous variables. *p*-values of less than 0.05 were considered statistically significant.

In addition, univariable and multivariable logistic regression analyses were used to assess factors associated with higher risk perception. The dependent variable was perceived exposure risk, dichotomized as high versus low. The independent variables included in the univariable analysis were gender, age group, faculty, current medical practice, years of professional experience, history of previous exposures, and the number of previous exposures. Variables with *p*-values less than 0.10 in the univariable analysis were considered for inclusion in the multivariable analysis [15]. For the univariable analysis, we report the odds ratio (OR), corresponding 95% confidence intervals (95% CIs) and *p*-values, while for multivariable analysis, we report the adjusted odds ratio (aOR), adjusted 95% confidence intervals (a95% CIs) and *p*-values. Multicollinearity among the independent variables was assessed using the variance inflation factor (VIF). Model fit was evaluated by comparing the multivariable model with the null model using the likelihood-ratio chi-square test, together with the Akaike information criterion (AIC) and Bayesian information criterion (BIC). Potentially influential observations were assessed using Cook’s distance, with values exceeding the 4/*n* threshold considered indicative of potential influence. The analyses were carried out in RStudio version 2026.08.0 Build 187 and in JASP (Version 0.98.1).

### 2.4. Ethical Considerations

This study was conducted in compliance with the student evaluation rules of the Carol Davila University of Medicine and Pharmacy, Bucharest, as included and approved in the internal activity plan for the 2021–2022 academic year of the Department of Epidemiology. Approval from the ethics committee of the Carol Davila University of Medicine and Pharmacy, Bucharest, was obtained to communicate the results in the form of scientific articles and presentations (approval number 9206). Participation was voluntary, with all data being processed in accordance with data protection regulations.

## 3. Results

*Study population:* Out of the total number of 911 respondents, 657 (72%) were students at the Faculty of Medicine, and 254 (28%) were students at the Faculty of Dental Medicine. The median age of the respondents was 24 years (IQR: 23–24); most of the respondents belonged to the 20–24-year-old age group (710; 78%), with statistically significant differences between groups. In addition, most respondents were female (651; 71%), reflecting the demographic pattern of the Romanian medical sector [16,17] (Table 1).

Of the 911 respondents, 352 (39%) reported participating in clinical practice or volunteering, with most being students at the Faculty of Dental Medicine (194; 55%). Clinical involvement was reported by 76% (194) of Dental Medicine students, compared with 24% (158) of Medicine students (*p* < 0.001). Among Medicine students, 36% (57) had more than 3 years of clinical experience, while only 16% (31) of Dental Medicine students reported more than 3 years of practice. The differences between the two groups of students are presented in Table 2.

Knowledge: Regarding knowledge, 119 respondents (13%) correctly identified all three principles of universal precautions, as previously defined, with no statistically significant differences among the two groups. In contrast, 881 (97%) of the respondents correctly identified all the measures included in the standard precautions, but only 222 (24%) identified the recommended sequence of PPE doffing, with differences between groups (<0.001). Half of the respondents (502; 55%) reported HBV as being associated with the highest risk of transmission after exposure, with a statistically significant number of Dental Medicine students recognizing the risk (152; 60% vs. 350; 53%, *p* = 0.003). However, significantly lower percentages of Dental Medicine students recognized an anti-HB antibody concentration higher than 10 mIU/mL as protective (173; 68% vs. 536; 82%; *p* < 0.001), and about half of them would induce bleeding in case of an exposure, either instinctively (54; 21% vs. 156; 24%) or because they consider it necessary (76; 30% vs. 105; 16%) (*p* < 0.001). Higher percentages of Medicine students compared with Dental Medicine students recognized the saline washing of the ocular mucosa after exposure as the correct conduit in case of exposure (627; 95% vs. 230; 91%; *p* = 0.005) and the importance of reporting the event to a supervisor for proper post-exposure management (636; 97% vs. 227; 89%; *p* < 0.001) (Table 3).

Practices: Over 60% (569; 62%) of respondents were aware of being vaccinated against hepatitis B, with significantly higher proportions of Medicine students (459; 70% vs. 110; 43%; *p* < 0.001), but with lower percentages of students reporting an antibody measurement at least once since they started practice (266; 29%), with statistically significant differences between groups. Over three-quarters of the respondents reported the use of PPE every time needed or most of the time (743; 82%), with differences between groups in terms of reported non-use and PPE availability at the facility level (*p* < 0.001) and higher percentage of Medicine students reporting. When discussing the habit of recapping needles, 157 (62%) of Dental Medicine students and 363 (55%) of Medicine students reported this habit, as they considered it essential for protecting those around (478; 92%) or when using the needle for the administration of medication to the same patient (42; 8%) (*p* < 0.001). Most respondents (836; 92%) recognized that yellow receptacles are recommended for disposal of used PPE, with a higher percentage of Dental Medicine students reporting not considering the receptacle important (14; 6% vs. 12; 2%; *p* = 0.012). In addition, most students (703; 77%) reported disposal of sharp instruments in the recommended receptacles, with no statistical differences among groups (Table 4). The reported reasons for wrong disposal of sharps were lack of attention (28; 39%), precipitation (19; 26%), lack of information on proper disposal (13; 18%), unavailability of receptacles (11; 15%) and considering it not important (1; 2%), with no statistically significant differences among groups (*p* = 0.2).

Past exposures: Ninety-three students (10%) reported at least one past exposure, with no statistically significant differences among groups (*p* = 0.2); 64 (69%) exposures were reported among females.

Most cases involved one percutaneous exposure (56; 60%), with the reported reasons for exposure being lack of attention (50; 54%), precipitation (45; 49%), tiredness (17; 18%), stress (14; 15%), lack of information (5; 5%) and lack of experience (2; 2%). Fifty-nine respondents (64%) reported the announcement of the event to their supervisor, with no statistically significant differences among groups (Table 5).

Risk perception: Statistically significantly higher percentages of Dental Medicine students reported perceived risk scores of 4 and 5, considered as high, compared to Medicine students (117; 46% vs. 202; 31%; *p* < 0.001). Univariable analysis showed Medicine students as having lower odds of reporting high perceived risk compared to Dental Medicine students (OR: 0.52; 95% CI: 0.39–0.70; *p* < 0.001). In contrast, higher odds of perceived high risk were associated with at least one past exposure (OR: 1.95; 95% CI: 1.27–3.01; *p* = 0.002), increasing with the number of past exposures (OR: 2.53; 95% CI: 1.27–5.05; *p* = 0.009). The final multivariable analysis model adjusted for gender, age group, faculty, and history of past exposure showed that being a Medicine student was associated with lower odds of perceived high risk (aOR: 0.54; 95% aCI: 0.39–0.73; *p* < 0.001). In contrast, reporting at least one past exposure was associated with higher odds of reporting high perceived risk (aOR: 1.94; 95% aCI: 1.25–3.00; *p* = 0.003) (Table 6). The multivariable logistic regression model was significantly better than the null model (likelihood-ratio χ^2^ (5) = 30.458, *p* < 0.001), with an AIC of 1160.512 and a BIC of 1189.393. No influential observations were identified, and no relevant multicollinearity was observed (VIF range: 1.005–1.102). Frequency of past exposure showed a *p*-value < 0.10 in the univariable analysis, but it was not included in the multivariable model because of collinearity with history of past exposures.

## 4. Discussion

Our university places a strong emphasis on delivering training tailored to students’ needs, with the goal of safeguarding their health and wellbeing. Regarding occupational exposure to blood and body fluids, which could create a medical and psychological burden, students have to receive regular training in accordance with the established regulations and procedures while undertaking practical work in healthcare settings [18]. Additionally, the concepts are extensively covered in the Epidemiology module, which takes place in the 4th year of study for the students of the Faculty of Dental Medicine and in the 6th year for the students of the Faculty of Medicine. Therefore, almost all (97%) students included in our study knew all the measures included in standard precautions. However, only 13% identified all three principles of universal precautions, which may represent a vulnerability, given that the patient’s medical history is not known or the patient, whether intentionally or unintentionally, does not communicate the fact that they have blood-borne diseases.

The correct sequence for removing PPE was known by a significantly higher percentage of Dental Medicine students. This is consistent with the fact that Dental Medicine students reported wearing protective equipment every time at a significantly higher percentage (73%) than Medicine students (34%). This may be due to the intensive practical dental activity they carry out and the fact that, during the COVID-19 pandemic, special attention was paid to the knowledge and application of personnel protection measures.

A significantly higher percentage of Dental Medicine students also knew that the highest risk after exposure is for HBV infection, but less than half (43%) reported knowing to be vaccinated against hepatitis B, and 81% of them had never measured their anti-HB antibody level. In contrast, a significantly higher percentage (70%) of students at the Faculty of Medicine knew their vaccination status and had measured their antibody levels at least once (33%), perhaps because they benefit from clinical training in hospitals, including in the infectious disease wards, facilitating their access to these services.

In Romania, the hepatitis B vaccine was introduced into the routine immunization schedule in 1995, with the first dose at birth. Subsequent efforts focused on catch-up vaccinations, particularly among 18-year-olds and students in medical schools. Therefore, all medical students in our group were eligible for hepatitis B vaccination [10,11]. In addition, for all unvaccinated healthcare workers, the hepatitis B vaccine has been fully reimbursed since December 2023 [19]. Previously, healthcare facilities were continuously required to assess the hepatitis B vaccination status of their employees and provide vaccination to those who were unprotected [9,10].

Almost half (45%) of the Medicine students were aware that used needles should not be recapped. A significantly lower percentage (38%) of Dental Medicine students stated the same thing, perhaps because in dental practice, there are situations where needles could be recapped. In such situations, mechanical devices allowing for safe recapping are recommended to prevent staff exposure [20].

It is worth noting that almost half of the students (52%) would recap used needles to avoid exposing others—but in fact, they would be exposing themselves.

In our study, 10% of students reported having had at least one past exposure. Although lower than the 27% and 59% reported in other single-institution studies, this figure should be interpreted cautiously, as under-reporting related to recall and social desirability biases cannot be excluded, as well differences in study designs [21,22].

An Australian study conducted over two decades ago, which included both general medicine and dentistry students, reported that 22% of medical students and 72% of dentistry students suffered at least one penetrating sharps injury [23].

Although, in our study, no statistically significant differences were demonstrated regarding the overall level of past exposures, we found that percutaneous exposure predominated in the group of Dental Medicine students (68% of total exposures), while in the case of Medicine students, there were also cases with multiple exposure: percutaneous, cutaneous and mucosal. These results reflect the particularities of the practical activities specific to each specialization.

Dental practitioners are known to have an increased risk of needlestick injuries, although data in the literature on the overall incidence of NSIs in this category are lacking. Previous studies considered the frequency of NSIs occurring in dental students to be alarmingly high, even in the context in which the phenomenon of under-reporting is well recognized [24,25].

The reasons involved in OEBBF are multiple and diverse, with our group of students reporting lack of attention, precipitation, tiredness, stress, lack of information and lack of experience, in accordance with data from the literature [21].

Only 64% of students who suffered an OEBBF correctly reported the event to a supervisor, with no significant differences between groups. Under-reporting of professional exposures among students is a generally recognized phenomenon that has been previously described in other large studies. For example, studies conducted in the US have shown that between 29% and 53% of students reported experiencing such an event [21,22,26].

On the other hand, a recent study conducted at Würzburg University, Germany, found that 81% of medical students’ NSIs were reported, with this high percentage being justified by the fact that the reporting procedure is included in the training curricula and counseling and screening are mandatory before starting practical training [21].

It is noteworthy that a significant percentage of students with past exposure (23% of Medicine students and 6% of Dental Medicine students) reported the event to a colleague. This aspect suggests that students have more confidence in communicating with peers who are at the same stage of training than with supervisors. It also shows the need to increase awareness, highlighting the legal obligation, as well as the benefits, of reporting an OEBBF directly to a supervisor [9,10].

This phenomenon underscores the importance of providing continuous training to all students and all staff categories on the prompt implementation of post-exposure prophylaxis and case management protocols, both for their own protection and to enable appropriate management that arises in the work team.

Training in the prevention and management of OEBBF must be adapted to new educational approaches and not limited to the theoretical area and clinical departments. It must also be carried out in simulation centers, where students learn to perform invasive procedures and the infectious risk is often neglected. In fact, it was shown that medical students who underwent practical training on simulators and objects were more prone to suffer from needlestick injuries [26].

In our study, we found that Dental Medicine students perceive a higher risk of exposure compared to their Medicine colleagues and have particular training needs, focusing on awareness of the importance of reporting and correct management of OEBBF, HBV infection prophylaxis, and medical waste management.

Additionally, our study highlighted that students who had at least one past exposure are more aware of exposure likelihood and reported a high perceived risk.

In spite of its limitations (namely, limited to a group of students from a single university; conducted in the 2021–2022 academic year; and self-reported data, which may introduce potential recall, social desirability, and under-reporting biases), our study remains highly relevant considering the large number of respondents, as well as the absence of more recent or comprehensive research on this topic. Moreover, considering our study objectives, it helped us to refine our educational materials and teaching strategies to better address the specific educational needs identified among the students.

From a broader perspective, our study provides a framework for evaluating educational needs at the university level and could serve as a model for similar assessments in other universities. Our findings emphasize the importance of ensuring that Medicine and Dental Medicine students receive comprehensive training on OEBBF prevention and reporting, ideally before the start of clinical practice. Students should always be regarded as healthcare workers in training and therefore exposed to comparable or even higher occupational risks. Strengthening collaboration among medical universities, healthcare facilities involved in students’ training, and public health authorities is necessary to ensure adequate training, sufficient resources for primary and secondary prophylaxis, and timely surveillance of OEBBF among students, thereby reducing their overall burden.

Nonetheless, our study opens future research directions, particularly with respect to the investigation of factors associated with percutaneous exposure, which was the most frequently reported type of occupational exposure in our study, to better inform targeted prevention strategies.

## 5. Conclusions

A low reporting frequency of past OEBBF was recorded among medical students included in our study. However, increasing efforts should be made to improve the knowledge and awareness of medical students regarding OEBBF risk and preventive measures before being exposed, starting from the beginning of their medical training. In addition, sustained efforts should be made to assess the vaccination status and the level of protection against hepatitis B, especially among Dental Medicine students. Nonetheless, students should be encouraged to immediately report any occupational exposure to blood to their supervisor for proper post-exposure management.

## Figures and Tables

**Table 1:** Demographic characteristics of the respondents, by faculty, at the Carol Davila University of Medicine and Pharmacy, Bucharest, Romania, 2021–2022.

Variable	Total N = 911 ^1^	Dental Medicine N = 254 ^1^	Medicine N = 657 ^1^	*p*-Value ^2^
Gender				0.5
Female	651 (71%)	186 (73%)	465 (71%)	
Male	260 (29%)	68 (27%)	192 (29%)	
Age	24.00 (23.00, 24.00)	22.00 (22.00, 23.00)	24.00 (24.00, 25.00)	<0.001
Age group (years)				<0.001
20–24	710 (78%)	244 (96%)	466 (71%)	
25–29	190 (21%)	7 (3%)	183 (28%)	
30+	11 (1%)	3 (1%)	8 (1%)	

^1^ n (%); median (IQR). ^2^ Pearson’s Chi-squared test; Wilcoxon rank-sum test; Fisher’s exact test.

**Table 2:** Distribution of respondents according to faculty and period since they started medical practice in a healthcare facility, Carol Davila University of Medicine and Pharmacy, Bucharest, Romania, 2021–2022.

Variable	Total N = 352 ^1^	Dental Medicine N = 194 ^1^	Medicine N = 158 ^1^	*p*-Value ^2^
Years of practice				<0.001
<1 year	129 (37%)	74 (38%)	55 (35%)	
1–3 years	135 (38%)	89 (46%)	46 (29%)	
>3 years	88 (25%)	31 (16%)	57 (36%)	

^1^ n (%). ^2^ Pearson’s Chi-squared test.

**Table 3:** Comparative distribution of the respondents according to faculty and responses to knowledge items, Carol Davila University of Medicine and Pharmacy, Bucharest, Romania, 2021–2022.

Variable	Total N = 911 ^1^	Dental Medicine N = 254 ^1^	Medicine N = 657 ^1^	*p*-Value ^2^
Universal precaution principles	119 (13%)	36 (14%)	83 (13%)	0.5
Standard precautions	881 (97%)	246 (97%)	635 (97%)	0.9
Correct PPE doffing sequence	222 (24%)	92 (36%)	130 (20%)	<0.001
Knowledge of the risk of viral transmission after exposure				0.003
HBV	502 (55%)	152 (60%)	350 (53%)	
HCV	151 (17%)	25 (10%)	126 (19%)	
HIV	258 (28%)	77 (30%)	181 (28%)	
Knowledge of the protective anti-HB antibody concentration	709 (78%)	173 (68%)	536 (82%)	<0.001
Bleeding stimulation				<0.001
Instinctively	210 (23%)	54 (21%)	156 (24%)	
Necessary	181 (20%)	76 (30%)	105 (16%)	
No	520 (57%)	124 (49%)	396 (60%)	
Proper conduit in case of ocular mucosa exposure	857 (94%)	230 (91%)	627 (95%)	0.005
The need to report the event to a supervisor	863 (95%)	227 (89%)	636 (97%)	<0.001

^1^ n (%). ^2^ Pearson’s Chi-squared test.

**Table 4:** Comparative distribution of the respondents according to faculty and responses to practices items, Carol Davila University of Medicine and Pharmacy, Bucharest, Romania, 2021–2022.

Variable	Total N = 911 ^1^	Dental Medicine N = 254 ^1^	Medicine N = 657 ^1^	*p*-Value ^2^
Known to be vaccinated against hepatitis B	569 (62%)	110 (43%)	459 (70%)	<0.001
Anti-HB antibody concentration measurement				<0.001
Once	226 (25%)	35 (14%)	191 (29%)	
Regular	40 (4%)	13 (5%)	27 (4%)	
Never	645 (71%)	206 (81%)	439 (67%)	
PPE use				<0.001
Every time	406 (45%)	186 (73%)	220 (34%)	
Most of the time	337 (37%)	58 (23%)	279 (42%)	
Rarely	86 (9%)	6 (2%)	80 (12%)	
Not available every time	82 (9%)	4 (2%)	78 (12%)	
Needle recapping				<0.001
No	391 (43%)	97 (38%)	294 (45%)	
Yes, for the same patient	42 (5%)	29 (11%)	13 (2%)	
Yes, to avoid exposing others	478 (52%)	128 (51%)	350 (53%)	
Disposal of used PPE				0.012
Yellow receptacle	836 (92%)	227 (89%)	609 (93%)	
Black receptacle	47 (5%)	13 (5%)	34 (5%)	
Considering it not important	26 (3%)	14 (6%)	12 (2%)	
Disposal of sharp instruments in receptacles other than those recomended				0.7
Never	703 (77%)	199 (78%)	504 (77%)	
I do not know	136 (15%)	38 (15%)	98 (15%)	
Yes	72 (8%)	17 (7%)	55 (8%)	

^1^ n (%). ^2^ Pearson’s Chi-squared test.

**Table 5:** Comparative distribution of the respondents reporting at least one past exposure according to faculty, Carol Davila University of Medicine and Pharmacy, Bucharest, Romania, 2021–2022.

Variable	Total N = 93 ^1^	Dental Medicine N = 31 ^1^	Medicine N = 62 ^1^	*p*-Value ^2^
Gender				0.4
Female	64 (69%)	23 (74%)	41 (66%)	
Male	29 (31%)	8 (26%)	21 (34%)	
Frequency of past exposure				0.9
Many times	34 (37%)	11 (35%)	23 (37%)	
Once	59 (63%)	20 (65%)	39 (63%)	
Type of exposure				0.052
Cutaneous	25 (27%)	6 (19%)	19 (31%)	
Mucous	9 (10%)	4 (13%)	5 (8%)	
Percutaneous	45 (48%)	21 (68%)	24 (39%)	
Cutaneous Mucous	3 (3%)	0 (0%)	3 (5%)	
Percutaneous Cutaneous	7 (8%)	0 (0%)	7 (11%)	
Percutaneous Mucous	1 (1%)	0 (0%)	1 (1%)	
Percutaneous Cutaneous Mucous	3 (3%)	0 (0%)	3 (5%)	
Number of reported exposure reasons				<0.001
1	66 (71%)	31 (100%)	35 (56%)	
2	18 (19%)	0 (0%)	18 (29%)	
3	5 (5%)	0 (0%)	5 (8%)	
4	4 (5%)	0 (0%)	4 (7%)	
Past exposure reporting				0.068
Supervisor	59 (64%)	20 (65%)	39 (63%)	
Colleague	16 (17%)	2 (6%)	14 (23%)	
No	18 (19%)	9 (29%)	9 (14%)	

^1^ n (%). ^2^ Pearson’s Chi-squared test.

**Table 6:** Univariable and multivariable analyses of factors associated with perceived exposure risk among students, Carol Davila University of Medicine and Pharmacy, Bucharest, Romania, 2021–2022.

Independent Variable	Univariable Analysis		Multivariable Analysis	
	OR (95% CI)	*p*-Value	aOR (a95% CI)	*p*-Value
Gender				
Female	Reference	Reference	Reference	Reference
Male	0.77 (0.56–1.04)	0.09	0.77 (0.56–1.05)	0.10
Age group (years)				
20–24	Reference	Reference	Reference	Reference
25–29	0.75 (0.53–1.05)	0.095	0.93 (0.64–1.33)	0.68
30+	0.65 (0.17–2.48)	0.530	0.67 (0.17–2.58)	0.56
Faculty				
Dental Medicine	Reference	Reference	Reference	Reference
Medicine	0.52 (0.39–0.70)	<0.001	0.54 (0.39–0.73)	<0.001
Medical practice				
No	Reference	Reference		
Yes	1.19 (0.90–1.58)	0.213		
Years of practice				
<1	Reference	Reference		
1–3	1.28 (0.78–2.11)	0.327		
>3	1.07 (0.61–1.88)	0.823		
Past exposures				
No	Reference	Reference	Reference	Reference
Yes	1.95 (1.27–3.01)	0.002	1.94 (1.25–3.00)	0.003
Frequency of past exposure				
None	Reference	Reference		
Once	1.68 (0.99–2.87)	0.055		
Many times	2.53 (1.27–5.05)	0.009		

## Data Availability

The raw data supporting the conclusions of this article will be made available by the authors on request.
